# Decreased lung function is associated with risk of developing non-alcoholic fatty liver disease: A longitudinal cohort study

**DOI:** 10.1371/journal.pone.0208736

**Published:** 2019-01-23

**Authors:** Jae-Uk Song, Yoonjung Jang, Si-Young Lim, Seungho Ryu, Won Jun Song, Christopher D. Byrne, Ki-Chul Sung

**Affiliations:** 1 Division of Pulmonary and Critical Care Medicine, Department of Internal Medicine, Kangbuk Samsung Hospital, Sungkyunkwan University School of Medicine, Seoul, Republic of Korea; 2 Center for Cohort Studies, Total Healthcare Center, Kangbuk Samsung Hospital, Sungkyunkwan University School of Medicine, Seoul, Republic of Korea; 3 Department of Occupational and Environmental Medicine, Kangbuk Samsung Hospital, Sungkyunkwan University School of Medicine, Seoul, Republic of Korea; 4 Department of Clinical Research Design & Evaluation, SAIHST, Sungkyunkwan University, Seoul, Republic of Korea; 5 Department of Critical Care Medicine, Kangbuk Samsung Hospital, Sungkyunkwan University School of Medicine, Seoul, Republic of Korea; 6 Nutrition and Metabolism Group, Southampton General Hospital, University of Southampton, Southampton, UK Southampton National Institute for Health Research, Biomedical Research Centre, Southampton General Hospital, University of Southampton, Southampton, United Kingdom; 7 Division of Cardiology, Department of Internal Medicine, Kangbuk Samsung Hospital, Sungkyunkwan University School of Medicine, Seoul, Republic of Korea; Universita degli Studi di Verona, ITALY

## Abstract

**Background:**

Decreased lung function is associated with non-alcoholic fatty liver disease (NAFLD), based on linking mechanisms such as insulin resistance and systemic inflammation However, its association with the risk of developing NAFLD is unclear. Our aim was to investigate whether baseline lung function is associated with incident NAFLD in middle-aged healthy Koreans.

**Methods:**

A cohort study of 96,104 subjects (mean age: 35.7 years) without NAFLD were followed up from 2002 to 2015. NAFLD was diagnosed by ultrasound after the exclusion of other possible causes of liver diseases. Baseline percent predicted forced expiratory volume in one second (FEV1%) and forced vital capacity (FVC%) were categorized in quartiles. Adjusted hazard ratios (aHR) and 95% confidence intervals (CIs) (using the highest quartile as reference) were calculated for incident NAFLD at follow-up, controlling for covariates and potential confounders.

**Results:**

During 579,714.5 person-years of follow-up, 24,450 participants developed NAFLD (incidence rate, 42.2 per 1,000 person-years). The mean follow-up period was 5.9±3.4 years. Regardless of smoking history, the risk for incident NAFLD increased with decreasing quartiles of FEV1 (%) and FVC (%) in a dose-response manner (*p* for trend<0.001). In never smokers, the aHRs (95% CIs) for incident NAFLD were 1.15 (1.08–1.21), 1.11 (1.05–1.18), and 1.08 (1.02–1.14) in quartiles 1–3 for FEV1 (%) and 1.12 (1.06–1.18), 1.11 (1.05–1.18), and 1.09 (1.03–1.15) in quartiles 1–3 for FVC (%), compared with the highest quartile reference. Similar inverse association was present in smoke-exposed subjects (aHR for incident NAFLD were 1.14, 1.21, 1.13 and 1.17, 1.11, 1.09 across FEV1(%) and FVC(%) quartile in increasing order, respectively).

**Conclusions:**

Reduced lung function was a risk factor for incident NAFLD in a large middle-aged Korean cohort with over half a million person-years of follow-up.

## Introduction

Non-alcoholic fatty liver disease (NAFLD) is characterized by the presence of hepatic steatosis [1] and may progress over time to cirrhosis and hepatic failure [2]. NAFLD is associated with various extrahepatic complications such as cardio-metabolic diseases, chronic kidney disease, and sarcopenia [3–7], mediating increased low-grade systemic inflammation [8,9] which play a causal role in the development of NAFLD [8]. Recently, the relationship between lung function and cardio-metabolic conditions has also been highlighted. Previous studies have demonstrated that decreased lung function is associated with an increase in low-grade inflammation [10] and increased risk of diabetes, cardiovascular disease, and metabolic syndrome [11,12]. Accordingly, there might be possibility linking decreased lung function to an increased risk of NAFLD, in the light of sharing inflammatory process. Therefore, we hypothesize that decreased lung function could be contributed to the development of NAFLD.

Although cross-sectional studies have suggested that reduced lung function measured at a single point in time is strongly associated with NAFLD [13–16], no longitudinal studies have investigated the role of baseline lung function in the development of incident NAFLD among subjects who are free of NAFLD at baseline. Using longitudinal follow-up data from a health screening examination program in South Korea in which it is possible to identify subjects with NAFLD using liver ultrasound data, we investigated whether baseline lung function was associated with incident NAFLD over 13 years of follow-up.

## Methods

### Study design and population

**Fig 1:**
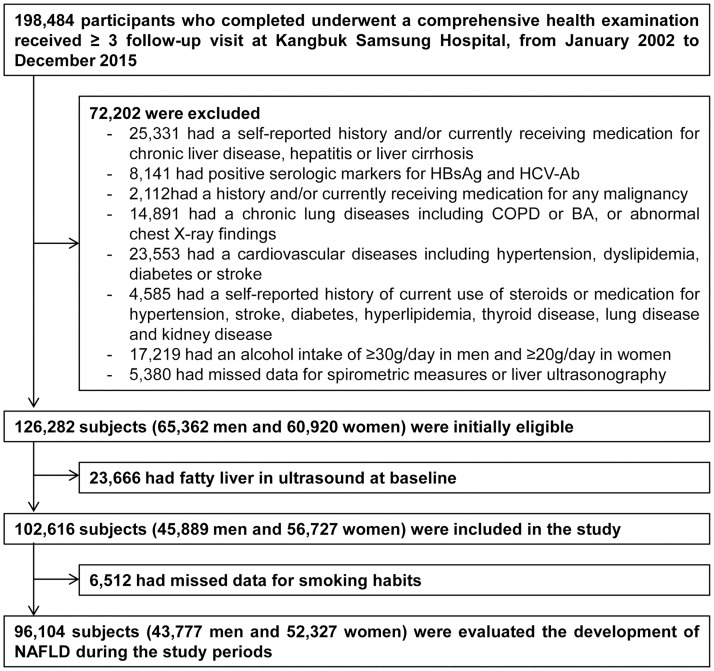
Flow chart of study participants.

The Kangbuk Samsung Health Study was a cohort study of South Koreans aged 18 years or older who underwent a comprehensive annual or biennial health examination at one of the Kangbuk Samsung Hospital Health Screening Centers located in Seoul and Suwon, South Korea, between 1 January 2002 and 31 December 2015. More than 80% of the participants were employees of various companies and local governmental organizations and their spouses. In South Korea, the Industrial Safety and Health Law requires employees to participate in annual or biennial health examinations, which are offered free of charge. The remaining participants voluntarily purchased screening exams at the health exam center. A total of 198,484 potential participants who completed a physical activity questionnaire and underwent a comprehensive health examination received at least three follow-up visits over the study period. The data from the first visit served as baseline data. Among these potential study subjects, we excluded participants that met the following criteria: a self-reported history and/or currently receiving medication for chronic liver disease (*n* = 25,331), including positive serologic markers for hepatitis B or C virus (*n* = 8,141); alcohol intake ≥30 g/day for men and ≥20 g/day for women (*n* = 17,219); history of malignancy (*n* = 2,112), cardiovascular-metabolic diseases or chronic pulmonary diseases including abnormal chest radiograph findings (*n* = 38,444); and currently receiving treatment with steroids or medication for diabetes, hyperlipidemia, or thyroid disease (*n* = 4,585). Specific details of comorbidities were not available because the medical history questionnaire only required yes/no responses. We also excluded participants with missing spirometric data or liver ultrasonography (*n* = 5,380). As some individuals met more than one of the above criteria for exclusion, 126,282 subjects were ultimately eligible for initial inclusion in the study. Of these, 23,666 (18.7%) had fatty liver on baseline ultrasound examination and an additional 6,512 subjects were excluded because of missing data for smoking habits. Finally, 96,104 subjects were included in the study (Fig 1).

The study was approved by the Institutional Review Board of the Kangbuk Samsung Hospital, which waived the requirement for informed consent as we used only de-identified data obtained as part of routine health screening examinations.

### Data collection, anthropometric measurements, and laboratory tests

At each visit, standardized self-administered questionnaires were used to acquire information regarding demographic characteristics, smoking status, drinking habits, regular exercise, medical history, current regular use of medications, and any clinical symptoms. Smoking habits were classified as follows: non-smokers, ex-smokers (no current smoking but regular smoking in the past), and current smokers (at least one cigarette per day). Alcohol history was considered positive if the subject had used alcohol in the past, even if they had stopped drinking. The weekly frequency of physical activity was also assessed and regular exercise was defined as ≥ 3 times/week.

Physical characteristics and serum biochemical parameters were measured. Height and weight were determined by trained nurses using automated instruments, with individuals wearing a lightweight hospital gown and no shoes. Height was measured to the nearest 1 mm using a stadiometer with the participant standing barefoot. Body weight was measured to the nearest 0.1 kg on a bioimpedance analyzer (InBody 3.0 and Inbody 720, Biospace Co., Seoul, Korea) that was validated for reproducibility and accuracy of body composition measurements [17]. Body mass index (BMI) was calculated as the weight in kilograms divided by the height in meters squared (kg/m^2^). Blood pressure was also measured by trained nurses with a standard sphygmomanometer following at least 5 minutes of seated rest.

Venous blood was collected from the antecubital vein after at least a 10-h fast. Methods for measuring serum levels of glucose, lipid profiles, liver enzymes, insulin, and high-sensitivity C-reactive protein (hsCRP) have been described previously [18]. Insulin resistance was assessed with the HOMA-IR equation: fasting blood insulin (μ U/ml) × fasting blood glucose (mmol/l)/22.5. The Laboratory Medicine Department at Kangbuk Samsung Hospital has been accredited and participates annually in inspections and surveys by the Korean Association of Quality Assurance for Clinical Laboratories.

### Ultrasuond examination and definition of NAFLD

Abdominal ultrasonography (Logic Q700 MR 3.5-MHz transducer; GE, Milwaukee, WI, USA) was conducted on all participants by 11 experienced radiologists who were unaware of the aims of the study and were blinded to the laboratory findings. Images were captured in a standard manner with the patient in the supine position with the right arm raised above the head. Fatty liver on ultrasonography was defined by an increase in echogenicity of the liver relative to the echogenicity of the renal cortex or spleen parenchyma [19]. The inter- and intra-observer reliability for fatty liver diagnosis was high (kappa statistics of 0.74 and 0.94 respectively) [20]. NAFLD was defined by the presence of fatty liver after the exclusion of excessive alcohol use (threshold of <30 g/d for men and <20 g/d for women) [21], or other identifiable causes of liver disease, as described in the exclusion criteria.

### Measurement of pulmonary function

Spirometry was performed as recommended by the American Thoracic Society [22] using the Vmax22 system (Sensor-Medics, Yorba Linda, CA). A bronchodilator was not administered prior to spirometry. The highest forced expiratory volume in 1 s (FEV1) and forced vital capacity (FVC) values from three or more tests with acceptable curves were used for further analyses. Spirometric values should be compared with the normal predictive values obtained from the same population using the same procedures, because FEV1 and FVC are affected by gender, age, height, weight and race [23]. In real practice, Morris equation, which is based on a study of American subjects [24], underestimate spirometric indices in koreans [25], because Asian people have larger lung volumes than Caucasians because they have shorter legs and longer upper bodies [26]. Therefore, the predicted values for FEV1 and FVC were calculated from the following equations obtained in a representative Korean population sample [25]:

$$\text{Predicted}\;\text{FVC}=-4.8434-\left(0.00008633\times \;{\text{age}}^{2}\left[\text{years}\right]\right)+\left(0.05292\times \text{height}\left[\text{cm}\right]\right)+\left(0.01095\times \text{weight}\left[\text{kg}\right]\right)$$

$$\text{Predicted}\;\text{FEV1}=-3.4132-\left(0.0002484\times \;{\text{age}}^{2}\left[\text{years}\right]\right)+\left(0.04578\times \text{height}\left[\text{cm}\right]\right).$$

The predicted FVC% (FVC [%]) and predicted FEV1% (FEV [%]) were calculated by dividing the FVC (L) and FEV1 (L) by the predicted FVC and FEV1, respectively.

### Statistical Analyses

Continuous variables were presented as the mean ± standard deviations or median and interquartile range, and categorical variables were presented as the number and percentage. The baseline data stratified by incident NAFLD at follow-up and quartiles of baseline ventilator function were compared using t-test or Mann–Whitney U test for two-group comparisons, and by one-way analysis of variance (ANOVA) or Kruskal-Wallis tests for comparison between quartiles. Chi-square test or Fisher’s exact test was used for categorical variables.

The outcome of interest was the development of incident NAFLD. Follow-up for each participant extended from the baseline exam until the development of NAFLD or the last health exam conducted prior to 31 December 2015, whichever came first. Incidence rates were calculated as the number of incident cases divided by person-years of follow-up. As we could establish that NAFLD had developed between two visits but could not determine the precise timing of NAFLD development, we used a parametric proportional hazards model to take interval censoring into account (stpm command in STATA) [27]. Using these models, the baseline hazard function was parameterized with restricted cubic splines in log time with four degrees of freedom.

The primary analysis was based on quartiles of baseline ventilator function. To exclude the potential confounding effects of smoking on the decline of lung function, the analyses were performed separately in smoke-exposed and smoke-naïve subjects. We estimated the adjusted hazard ratios (aHR) with 95% confidence intervals (CI) for incident NAFLD in quartiles 1–3 of FVC (%) or FEV1 (%) at baseline, with the highest (4^th^) quartile as the reference group. Logistic regression analyses were adjusted for baseline potential demographics, including age, sex, BMI, alcohol intake, smoking, exercise, education level, center, and year of test. We used a continuous variable with the number of quartiles and tested its statistical significance in the regression models. We assessed the proportional hazards assumption by examining graphs of estimated log (−log) survival. We also conducted dose–response analyses. We estimated the aHR with 95% CI associated with an increase of 1 (%) of ventilator function parameters at baseline using lung function as a continuous variable in the regression models. All tests were two-sided, and statistical significance was defined as a *p* value<0.05 (two-tailed). Data were analyzed using STATA version 13.0 (StataCorp LP, College Station, TX).

## Results

Table 1 summarizes the baseline characteristics of the enrolled subjects (*n* = 102,616). Mean age was 35.7 years and mean BMI was 22.0 kg/m^2^. Compared with the reference group, subjects in the incident NAFLD group were more likely to be men, to have smoked, and to drink alcohol, and had higher blood pressure, and higher levels of fasting glucose, total cholesterol, triglycerides, LDL-C, hepatic enzymes, insulin, HOMA-IR, and hsCRP and lower levels of HDL-C (Table 1). The mean FEV1 (%) and FVC (%) values were 86% and 85%. The baseline values of FVC (%) and FEV1 (%) in the incident NAFLD group were significantly lower than in the reference group. Furthermore, subjects in the incident NAFLD group showed a greater decline in both FEV1 (%) and FVC (%) over the study period (Table 1).

**Table 1:** Baseline characteristics according to the change in fatty liver status over the follow-up period.

Variables	Overall	No NAFLD (Reference)	Incident NAFLD	p value
**Number of subjects**	102,616	77,091 (75.1)	25,525 (24.9)	
**Age (years)**	35.7±6.2	35.6±6.2	36.1±6.1	<0.001
**Sex (male) (%)**	45,889 (44.7)	28,363 (36.8)	17,526 (68.7)	<0.001
**Smoking status (%) (n = 96,104)**				<0.001
** Non-smoker**	64,597 (67.2)	52,183 (72.8)	12,414 (50.8)	
** Former smoker**	12,295 (12.8)	8,172 (11.4)	4,123 (16.8)	
** Current smoker**	19,212 (20.0)	11,299 (15.8)	7,913 (32.4)	
**Median pack-years of smoking (IQR)**	0 (0–1.2)	0 (0–0)	0 (0–7.5)	<0.001
**Alcohol intake (%) (n = 95,544)**	21,574 (22.6)	13,721 (19.3)	7,853 (32.1)	<0.001
**Regular exercise (%) (n = 101,349)**	14,143 (14.0)	10,523 (13.8)	3,620 (14.4)	0.030
**High education (%) (n = 70,862)**	55,995 (79.0)	41,541 (78.7)	14,454 (80.0)	<0.001
**Systolic BP (mmHg)**	109±11	108±11	111±10	<0.001
**Diastolic BP (mmHg)**	70±8	69±8	72±8	<0.001
**BMI (kg/m**^**2**^**)**	22.0±2.6	21.5±2.4	23.6±2.4	<0.001
**Laboratory findings**				
** Fasting glucose (mg/dl)**	90.7±7.9	90.2±7.7	92.0±8.1	<0.001
** Total cholesterol (mg/dl)**	185.9±31.3	183.4±30.7	193.5±31.9	<0.001
** LDL-C (mg/dl)**	106.8±27.1	104.0±26.4	115.3±27.3	<0.001
** HDL-C (mg/dl)**	58.6±13.1	60.3±13.1	53.6±11.5	<0.001
** Triglyceride (mg/dl)**	83 (62–115)	77 (59–103)	107 (78–148)	<0.001
** AST (U/l)**	20 (17–23)	20 (17–23)	21 (18–25)	<0.001
** ALT (U/l)**	17 (13–22)	16 (12–21)	21 (16–28)	<0.001
** GGT (U/l)**	15 (10–22)	13 (10–20)	20 (13–30)	<0.001
** Insulin (mIU/ml)**	6.6 (4.6–8.6)	6.3 (4.4–8.3)	7.4 (5.5–9.5)	<0.001
** HOMA-IR**	1.46 (1.02–1.95)	1.40 (0.96–1.87)	1.66 (1.23–2.18)	<0.001
** hsCRP (mg/l)**	0.03 (0.01–0.07)	0.03 (0.01–0.06)	0.05 (0.02–0.09)	<0.001
**Baseline spirometric values**				
** Predicted FEV1 (%)**	86.3±16.5	91.3±16.7	84.7±16.1	<0.001
** Predicted FVC (%)**	85.4±14.9	89.4±14.8	84.1±14.7	<0.001
** FEV1/FVC ratio**	87±8	87±8	86±7	<0.001
** Obstructive pattern (FEV1/FVC <70%)**	1,948 (1.9)	1,471 (1.9)	477 (1.9)	0.689
**Percent change of spirometric values at time of NAFLD development or final examination**				
** Predicted FEV1 (%)**	-2.2±13.1	-1.4±12.8	-4.6±13.7	<0.001
** Predicted FVC (%)**	-0.4±11.5	0.2±11.4	-2.3±11.7	<0.001

ALT, alanine aminotransferase; AST, aspartate aminotransferase; BMI, body mass index; BP, blood pressure; FEV1 (%) predicted, percent predicted forced expiratory volume in 1 s; FVC (%) predicted, percent predicted forced vital capacity; GGT, gamma-glutamyltransferase; HDL-C, high-density lipoprotein cholesterol; hsCRP, high-sensitivity C-reactive protein; HOMA-IR, homeostasis model assessment of insulin resistance; LDL-C, low-density lipoprotein cholesterol; NAFLD, non-alcoholic fatty liver disease. Values represent mean±standard deviation or median and interquartile range or frequencies (number of subjects) with percentages. We recorded subject numbers with available clinical parameters. Unless otherwise indicated, available subject number was 102,616

When analyzed by quartiles of FEV1 (%) and FVC (%) level in smoke-exposed subjects or never smokers (Tables 2 and 3, respectively), both smoke-exposed and never smokers with lower FVC (%) or FEV1 (%) were less likely to drink and had higher BMIs. With respect to metabolic parameters, the subjects in the higher FVC (%) or FEV1 (%) quartiles had lower blood pressure, lower levels of LDL cholesterol, and triglyceride, and higher levels of HDL cholesterol, regardless of smoking habits. In contrast to never smokers, there was no association between FVC (%) or FEV1 (%) levels and exercise in smoke-exposed subjects. Additionally, levels of HOMA IR and insulin did not reach statistical significance across FVC (%) quartiles in smoke-exposed subjects.

**Table 2:** Comparisons between Four Groups according to Initial Spirometric values in smoke-exposed subjects without NAFLD at baseline.

Variables	Over all	Predicted FEV1 (%) quartiles					Predicted FVC (%) quartiles				
		1^st^ Q (≤78%)	2^nd^ Q (79–86%)	3^rd^ Q (87–94%)	4^th^ Q (≥95%)	p value	1^st^ Q (≤78%)	2^nd^ Q (79–85%)	3^rd^ Q (86–93%)	4^th^ Q (≥94%)	p value
**Number of participants**	31,507	3,625 (11.5)	5,536 (17.6)	8,423 (26.7)	13,923 (44.2)		3,408 (10.8)	6,113 (19.4)	8,600 (27.3)	13,386 (42.5)	
**Age (years)**	35.9±6.2	35.4±6.6	35.9±6.4	35.89±6.1	36.2±6.1	<0.001	36.2±7.2	36.0±6.4	35.8±6.1	35.9±5.9	0.133
**Sex (male) (%)**	28,649 (90.9)	2,086 (57.5)	4,818 (87.0)	8,082 (96.0)	13,663 (98.1)	<0.001	1,941 (57.0)	5,396 (88.3)	8,207 (95.4)	13,105 (97.9)	<0.001
**Smoking status (%)**						<0.001					<0.001
** Former smoker (%)**	12,295 (39.0)	1,651 (45.5)	2,260 (40.8)	3,207 (38.1)	5,177 (37.2)		1,592 (46.7)	2,568 (42.0)	3,344 (38.9)	4,791 (35.8)	
** Current smoker (%)**	19,212 (61.0)	3,274 (90.3)	4,476 (80.9)	6,016 (74.4)	5,446 (39.1)		3,116 (91.4)	4,745 (77.6)	6,056 (70.4)	5,295 (39.6)	
**Alcohol intake (%) (n = 30,782)**	14,314 (46.5)	1,157 (33.5)	2,418 (45.0)	3,932 (47.7)	6,807 (49.7)	<0.001	1,041 (32.1)	2,636 (44.2)	4,080 (48.5)	6,557 (49.8)	<0.001
**Regular exercise (%) (n = 31,340)**	4,325 (13.8)	488 (13.6)	742 (13.5)	1,140 (13.6)	1,955 (14.32)	0.199	476 (14.1)	824 (13.6)	1,167 (13.7)	1,858 (14.0)	0.711
**High education (%) (n = 22,248)**	18,755 (84.3)	1,966 (80.8)	3,131 (84.6)	4,802 (85.6)	8,856 (84.3)	0.006	1,812 (80.1)	3,305 (84.3)	4,939 (85.1)	8,699 (84.8)	<0.001
**SBP (mmHg)**	112±10	113±10	112±10	112±10	109±11	<0.001	113±10	112±10	111±10	109±11	<0.001
**DBP (mmHg)**	72±7	73±7	72±7	72±8	70±8	<0.001	73±7	72±7	72±7	70±8	<0.001
**BMI (kg/m**^**2**^**)**	22.9±2.5	23.2±2.4	23.0±2.4	22.8±2.6	21.9±2.7	<0.001	23.1±2.3	23.0±2.5	22.9±2.6	220.1±2.8	<0.001
**Fasting glucose (mg/dl)**	91.5±8.0	91.4±8.0	91.7±8.2	91.8±8.1	90.7±8.0	0.032	91.6±7.8	91.9±8.0	91.4±8.2	90.2±8.4	<0.001
**Total cholesterol (mg/dl)**	190.6±31.5	192.2±31.5	190.7±31.5	189.5±31.8	185.7±30.9	<0.001	191.3±31.5	190.3±31.3	190.8±31.8	187.9±31.8	<0.001
**LDL-C (mg/dl)**	112.6±27.5	113.7±27.0	113.7±27.6	112.2±28.1	106.9±57.5	<0.001	113.3±27.1	113.0±27.6	113.2±28.0	108.0±27.8	<0.001
**HDL-C (mg/dl)**	54.6±11.9	54.3±11.3	53.9±11.6	54.4±12.1	57.6±13.5	<0.001	54.2±11.3	54.1±11.8	54.2±11.8	57.8±13.6	<0.001
**Triglyceride (mg/dl)**	102 (76–141)	104 (78–142)	105 (77–143)	101 (74–141)	89 (66–128)	<0.001	104 (77–141)	104 (77–142)	104 (76–144)	90 (65–128)	<0.001
**AST (U/l)**	21 (18–25)	22 (19–25)	22 (18–25)	21 (18–25)	20 (17–24)	<0.001	22 (19–25)	21 (18–25)	21 (18–25)	21 (18–24)	<0.001
**ALT (U/l)**	21 (16–27)	21 (17–28)	21 (16–28)	20 (15–27)	18 (13–24)	<0.001	21 (16–28)	21 (16–28)	21 (16–28)	18 (13–25	<0.001
**GGT (U/l)**	21 (15–32)	22 (16–31)	23 (16–33)	21 (15–33)	17 (12–27)	<0.001	22 (16–32)	22 (16–33)	22 (15–33)	17 (12–28)	<0.001
**Insulin (mIU/ml)**	6.6 (4.8–8.5)	6.6 (5.0–8.4)	6.6 (4.7–8.6)	6.7 (4.7–8.7)	6.6 (4.7–8.6)	0.001	6.6 (4.9–8.4)	6.5 (4.6–8.5)	6.7 (4.8–8.7)	6.6 (4.8–8.8)	0.812
**HOMA-IR (mg/l)**	1.48 (1.06–1.94)	1.47 (1.09–1.92)	1.48 (1.04–1.96)	1.50 (1.04–1.98)	1.45 (1.02–1.95)	<0.001	1.47 (1.09–1.91)	1.47 (1.03–1.96)	1.50 (1.05–1.97)	1.45 (1.03–1.98)	0.133
**hsCRP (mg/l)**	0.04 (0.01–0.08)	0.04 (0.01–0.08)	0.04 (0.02–0.08)	0.04 (0.02–0.09)	0.03 (0.01–0.08)	0.057	0.04 (0.01–0.08)	0.04 (0.02–0.08)	0.04 (0.02–0.09)	0.03 (0.01–0.08)	0.002

ALT, alanine aminotransferase; AST, aspartate aminotransferase; BMI, body mass index; BP, blood pressure; FEV1 (%) predicted, percent predicted forced expiratory volume in 1 s; FVC (%) predicted, percent predicted forced vital capacity; GGT, gamma-glutamyltransferase; HDL-C, high-density lipoprotein cholesterol; hsCRP, high-sensitivity C-reactive protein; HOMA-IR, homeostasis model assessment of insulin resistance; LDL-C, low-density lipoprotein cholesterol. Values represent mean±standard deviation or median and interquartile range or frequencies (number of subjects) with percentages. We recorded subject numbers with available clinical parameters. Unless otherwise indicated, available subject number was 31,507.

**Table 3:** Comparisons between Four Groups according to Initial Spirometric values in never smokers without NAFLD at baseline.

Variables	Overall	Predicted FEV1 (%) quartiles					Predicted FVC (%) quartiles				
		1^st^ Q (≤79%)	2^nd^ Q (80–88%)	3^rd^ Q (89–95%)	4^th^ Q (≥96%)	p value	1^st^ Q (≤78%)	2^nd^ Q (79–85%)	3^rd^ Q (86–95%)	4^th^ Q (≥96%)	p value
**Number of subjects**	64,597	27,709 (42.9)	14,804 (22.9)	10,234 (15.8)	11,850 (18.3)		27,571 (42.7)	14,910 (23.1)	10,698 (16.6)	11,418 (17.7)	
**Age (years)**	35.6±6.1	35.2±5.8	35.8±6.0	35.8±6.2	36.4±6.6	<0.001	35.5±6.1	40.0±6.0	35.6±6.0	36.0±6.2	<0.001
**Sex (men) (%)**	15,128 (23.4)	1,018 (3.7)	2,598 (17.6)	4,477 (43.8)	7,035 (59.4)	<0.001	1,016 (3.7)	3,130 (21.0)	4,606 (43.1)	6,376 (55.8)	<0.001
**Alcohol intake (%) (n = 60,257)**	6,327 (10.5)	1,319 (5.2)	1,260 (9.3)	1,519 (15.7)	2,229 (19.3)	<0.001	1,285 (5.1)	1,364 (9.9)	1,603 (15.8)	2,075 (18.8)	<0.001
**Regular exercise (%) (n = 64,224)**	8,285 (12.9)	3,102 (11.3)	1,944 (13.2)	1,452 (14.3)	1,787 (15.2)	<0.001	3,210 (11.7)	1,948 (13.1)	1,449 (13.6)	1,678 (14.8)	<0.001
**High education (%) (n = 45,087)**	34,537 (76.6)	14,181 (75.6)	7,318 (75.1)	5,440 (77.0)	7,598 (79.7)	<0.001	13,958 (74.8)	7,329 (75.8)	5,789 (78.0)	7,461 (79.9)	<0.001
**SBP (mmHg)**	107±11	111±10	109±11	107±11	105±11	<0.001	110±10	109±11	107±11	105±11	<0.001
**DBP (mmHg)**	68±8	71±8	70±8	68±8	67±8	<0.001	71±8	70±8	68±8	67±8	<0.001
**BMI (kg/m**^**2**^**)**	21.6±2.5	22.5±2.4	22.2±2.4	21.7±2.4	21.1±2.4	<0.001	22.3±2.4	22.1±2.4	21.7±2.4	21.1±2.5	<0.001
**Fasting glucose (mg/dl)**	90.1±7.7	91.5±7.7	90.9±7.8	90.1±7.7	89.2±7.7	<0.001	91.3±7.6	91.1±7.7	90.36±7.7	89.1±7.7	<0.001
**Total cholesterol (mg/dl)**	183.8±30.9	186.6±31.1	185.3±31.6	183.3±30.6	182.2±30.7	<0.001	185.2±30.9	184.5±31.0	183.4±31.0	183.1±30.8	<0.001
**LDL-C (mg/dl)**	104.1±26.4	108.5±26.7	107.0±27.2	103.7±25.9	101.5±25.9	<0.001	107.5±26.6	106.5±26.7	103.9±26.4	101.9±25.9	<0.001
**HDL-C (mg/dl)**	60.4±13.1	57.3±12.2	58.7±12.8	60.6±13.2	62.1±13.2	<0.001	57.6±12.2	58.3±12.7	60.3±13.2	62.3±13.2	<0.001
**Triglyceride (mg/dl)**	76 (58–102)	85 (64–115)	79 (61–107)	74 (57–100)	72 (56–96)	<0.001	83 (63–113)	80 (61–108)	75 (58–101)	72 (56–96)	<0.001
**AST (U/l)**	19 (17–23)	21 (18–24)	20 (17–23)	19 (16–22)	19 (16–22)	<0.001	20 (17–24)	20 (17–23)	19 (17–22)	19 (16–22)	<0.001
**ALT (U/l)**	15 (12–20)	18 (14–24)	16 (13–22)	15 (12–19)	14 (11–18)	<0.001	18 (14–23)	16 (13–22)	15 (12–20)	14 (11–18)	<0.001
**GGT (U/l)**	13 (9–18)	16 (11–22)	14 (10–21)	12 (9–17)	11 (9–15)	<0.001	15 (11–22)	14 (10–21)	12 (9–17)	11 (9–15)	<0.001
**Insulin (mIU/ml)**	6.8 (4.8–8.9)	7.2 (5.4–9.23)	6.8 (4.8–8.8)	6.7 (4.6–8.9)	6.6 (4.6–8.7)	<0.001	7.2 (5.4–9.2)	6.78 (4.8–8.9)	6.72 (4.6–8.8)	6.58 (4.6–8.7)	<0.001
**HOMA-IR**	1.49 (1.04–1.99)	1.60 (1.20–2.11)	1.51 (1.06–2.01)	1.49 (1.01–1.99)	1.44 (0.99–1.93)	<0.001	1.61 (1.21–2.10)	1.52 (1.06–2.01)	1.49 (1.01–1.99)	1.43 (0.98–1.93)	<0.001
**hsCRP (mg/l)**	0.03 (0.01–0.06)	0.03 (0.01–0.06)	0.03 (0.01–0.06)	0.03 (0.01–0.06)	0.03 (0.01–0.06)	<0.001	0.03 (0.01–0.06)	0.03 (0.01–0.06)	0.03 (0.01–0.06)	0.03 (0.01–0.06)	0.003

ALT, alanine aminotransferase; AST, aspartate aminotransferase; BMI, body mass index; BP, blood pressure; FEV1 (%) predicted, percent predicted forced expiratory volume in 1 s; FVC (%) predicted, percent predicted forced vital capacity; GGT, gamma-glutamyltransferase; HDL-C, high-density lipoprotein cholesterol; hsCRP, high-sensitivity C-reactive protein; HOMA-IR, homeostasis model assessment of insulin resistance; LDL-C, low-density lipoprotein cholesterol.

**Fig 2:**
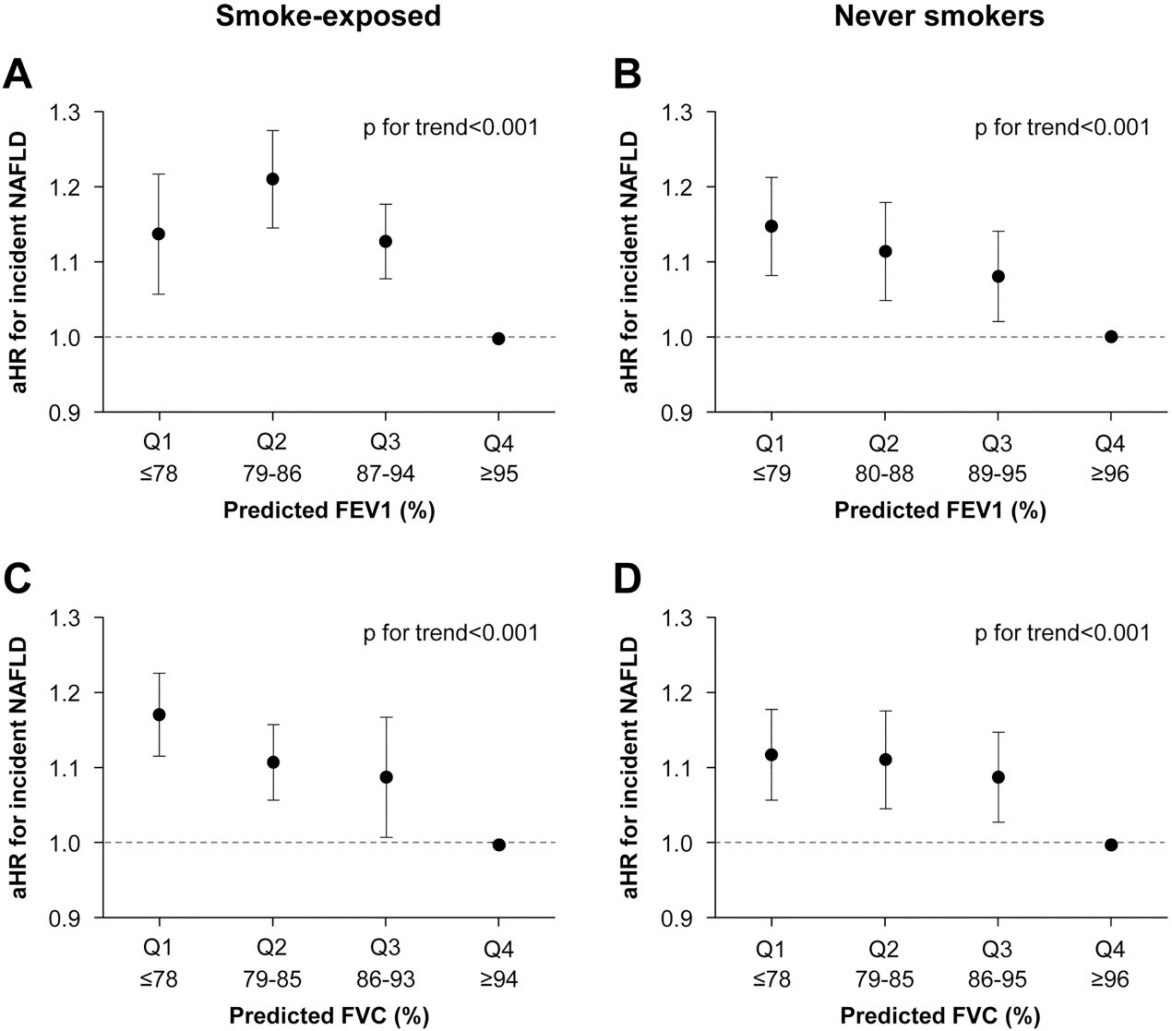
Multivariable-adjusted hazard ratios for incident nonalcoholic fatty liver disease according to quartile of lung function. Smoke-exposed subjects (A,C) and never smokers (B,D) were dived into quartiles according to baseline precentage of predcited values (% predicted) for FEV1 or FVC. Rregardless of smoking status, adjusted hazard ratios for incident NAFLD increased with decreasing quartiles of FEV1 (%) (A,B) and FVC (%) (C,D) in a dose-response manner (*p* for trend<0.001). The reference values was set at the highest quartile of FEV1(%) and FVC(%). Models was adjusted for potential covariates and metabolic laboratory markers including age, sex, BMI, alcohol intake, smoking, exercise, education level, center, year of test. aHR, adjusted hazard ratio; FEV1, forced expiratory volume in 1s; FVC, forced vital capacity; NAFLD, nonalcoholic fatty liver disease.

Table 4 shows the risk of developing NAFLD according to baseline FEV1 (%) and FVC (%) stratified by smoking habit. We identified 24,450 incident cases of NAFLD during 579,714.5 person-years of follow-up (incidence rate, 42.2 per 1000 person-years). The mean±SD follow-up period was 5.9±3.4 years. We analyzed the relationships between baseline spirometric values and incident NAFLD after adjusting for all potential confounding parameters at baseline. Regardless of smoking habit, a low baseline FEV1 (%) and FVC (%) was strongly associated with incident NAFLD. Compared with the highest quartile (the reference group) of FEV1 (%) at baseline, the aHRs (95% CIs) for incident NAFLD in quartiles 1–3 were 1.14 (1.06–1.22), 1.21 (1.15–1.28), and 1.13 (1.08–1.18) in smoke-exposed subjects and 1.15 (1.08–1.21), 1.11 (1.05–1.18), and 1.08 (1.02–1.14) in never smokers, respectively. Similarly, the lowest quartile of FVC (%) was also associated with a higher risk of developing NAFLD (aHR = 1.17 [1.12–1.23] in smoke-exposed subjects and aHR = 1.12 [1.06–1.18] in never smokers), compared with the highest quartile (Table 4 and Fig 2). The aHR for incident NAFLD associated with a 1% decrease when FEV1 (%) was introduced as a continuous variable in regression models was 1.07 (1.05–1.09) in smoke-exposed subjects and 1.003 (1.002–1.004) in never smokers. With respect to each 1% decrease in FVC (%), the aHR for incident NAFLD was 1.005 (1.003–1.006) in smoke-exposed subjects and 1.003 (1.001–1.004) in never smokers.

**Table 4:** Development of non-alcoholic fatty liver by quartiles of baseline spirometry values in smoke-exposed and never-smoker subjects.

Pulmonary function	Person-years	Number of incident cases	Incidence rate (per 1,000 person-years)	Multivariable-adjusted HR (95% CI)^a^
**Predicted FEV1 (%)**				
** Smoke-exposed (n = 31,507)**				
** 1**^**st**^ **Q (≤78%)**	53,649.23	3,771	70.29	1.14 (1.06–1.22)
** 2**^**nd**^ **Q (79–86%)**	45,657.31	3,298	72.23	1.21 (1.15–1.28)
** 3**^**rd**^ **Q (87–94%)**	39,121.25	2,637	67.41	1.13 (1.08–1.18)
** 4**^**th**^ **Q (≥95%)**	40,657.96	2,330	57.31	Reference
** p for trend**				<0.001
**Per 1% decrease in FEV1 (%)**				1.005 (1.004–1.006)
** Never smokers (n = 64,597)**				
** 1**^**st**^ **Q (≤79%)**	76,905.34	3,419	44.46	1.15 (1.08–1.21)
** 2**^**nd**^ **Q (80–88%)**	61,366.13	2,397	39.06	1.11 (1.05–1.18)
** 3**^**rd**^ **Q (89–95%)**	87,966.84	2,648	30.10	1.08 (1.02–1.14)
** 4**^**th**^ **Q (≥96%)**	174,390.45	3,950	22.65	Reference
** p for trend**				<0.001
**Per 1% decrease in FEV1 (%)**				1.003 (1.002–1.004)
**Predicted FVC (%)**				
** Smoke-exposed (n = 31,507)**				
** 1**^**st**^ **Q (≤78%)**	33,090.79	2,398	72.47	1.17 (1.12–1.23)
** 2**^**nd**^ **Q (79–85%)**	46,594.34	3,364	72.20	1.11 (1.06–1.16)
** 3**^**rd**^ **Q (86–93%)**	79,319.83	5,283	66.60	1.09 (1.01–1.17)
** 4**^**th**^ **Q (≥94%)**	20,080.79	991	49.35	Reference
** p for trend**				<0.001
**Per 1% decrease in FVC (%)**				1.005 (1.003–1.006)
** Never smokers (n = 64,597)**				
** 1**^**st**^ **Q (≤78%)**	74,595.42	3,119	41.811	1.12 (1.06–1.18)
** 2**^**nd**^ **Q (79–85%)**	64,612.78	2,493	38.58	1.11 (1.05–1.18)
** 3**^**rd**^ **Q (86–95%)**	88,805.63	2,768	31.17	1.09 (1.03–1.15)
** 4**^**th**^ **Q (≥96%)**	172,614.93	4,034	23.37	Reference
** p for trend**				<0.001
**Per 1% decrease in FVC1 (%)**				1.003 (1.001–1.004)

FEV1% predicted, percent predicted forced expiratory volume in 1 s; FVC% predicted, percent predicted forced vital capacity; HR, Hazard ratio.

^a^Estimated from parametric proportional hazards model adjusted for potential covariates and metabolic laboratory markers including age, sex, BMI, alcohol intake, smoking, exercise, education level, center, year of test.

## Discussion

The novel result of our study was that decreased FEV1 (%) and FVC (%) were independently associated with incident NAFLD over 13 years of follow-up. To the best of our knowledge, our study is the first to describe the association between low levels of lung function at baseline and incident NAFLD (diagnosed by liver ultrasound) in subjects without liver fat by ultrasound examination at baseline.

In the current study, 24,450 subjects developed NAFLD during 579,714.5 person-years of follow-up, which represents an incidence rate of 42.2 per 1000 person-years and is consistent with previous data (31–86 per 1,000 person-years) [28]. The baseline prevalence of NAFLD was approximately 18.7%; this is a little lower than previously reported (36.4–47.8%) [13, 15, 16] but the Korean population is not as obese as populations in other parts of the world. In a previous cross-sectional Korean study investigating associations between lung function and NAFLD, NAFLD was determined using only the hepatic steatosis index or aminotransferase levels [14]. Because aminotransferase levels can be normal in individuals with NAFLD [29] and have poor sensitivity and specificity for identifying NAFLD, it is possible that the prevalence of NAFLD in that study was imprecise [14] The prevalence of NAFLD in our study may be lower because the mean age of enrolled subjects was younger (36 years) than in previous studies (>47 years) [13,15,16]. Additionally, some subjects in previous studies had diabetes (7.4–8.1%), hypertension (11.8–28.1%), or metabolic syndrome (>50%) [13,15,16], all of which are associated with an increased risk of NAFLD.

Our results support a close link between NAFLD and lung function impairment including COPD severity from previous studies [13,15,16,30–33]. We also demonstrated that the increased risk of NAFLD with decreased lung function is irrespective of whether the subjects were smokers or never smokers. Furthermore, emerging evidence has shown that both NAFLD and impaired lung function were commonly associated with cardio-metabolic comorbidities [5,34–36]. Therefore, NAFLD and impaired lung function could be associated not by chance but by pathobiological necessity [37]. However, our results should be interpreted with caution, because of this modest effect of baseline lung function on the development of NAFLD. In the actual population, various factors such as life style behaviors and metabolic comorbidities contributed more to the development of NAFLD [38,39]. Furthermore, lung function decline rate was not associated with NAFLD itself in recent longitudinal analysis [31], although some subjects in this study had a medical history of metabolic syndrome (9.4%), hypertension (14%), and diabetes (4.1%) which can be a risk of NAFLD and decreased lung function [11,35,36,40]. Considering the results of this study and ours, decreased lung function is strongly associated with NAFLD, but additional studies should be needed to clarify their precise interrelationship, especially on the issue whether NAFLD itself affects lung function.

In the present study, the risk of incident NAFLD was interestingly approximately the same in smoke exposed subjects and non-smokers, suggesting that decreased lung function itself rather than its etiology such as smoking increase the risk of incident NAFLD. The influence of smoking on development of NAFLD is controversial. Some studies reported that smoking was a risk factor for NAFLD [41,42], while another study expresses a conflicting results [43,44]. In recent meta-analysis regarding this controversy, interestingly, there was an association between smoking and NAFLD in former smokers and passive smokers, whereas there was not any correlation in current smokers [45]. An increase in body weight and BMI as a consequence of cessation of smoking [46] and higher concentration of harmful chemicals in side stream smoke than main stream smoke [47] could be an explanation for the development of NAFLD in former smoker and passive smokers, respectively. Of note, there is a body of evidence that smoking is an important factor for several metabolic disturbances [48] which are closely related to NAFLD [35]. Finally, smoking itself may act as a cofactor but not as a direct independent factor for NAFLD. Given indirect role of smoking for incident NAFLD, it seems to have a similar risk of incident NAFLD, regardless of smoking status, although the exact proportion of passive smokers among never smokers could not be evaluated in the current study.

The underlying mechanisms relating reduced lung function to development of NAFLD remain unclear. However, it is plausible that insulin resistance plays a role because it is closely associated with both NAFLD [49] and reduced lung function [50]. Interestingly, liver steatosis is also associated with insulin resistance in skeletal muscle [51], and decreased muscle mass is associated with an increased risk of NAFLD [7]. Insulin resistance in skeletal muscle reduces glucose utilization and induces abnormal fat metabolism, which may impair mitochondrial ATP production and reduce skeletal muscle strength [51,52]. As forced respiration during spirometry requires respiratory skeletal muscle contraction, a decline of lung function could be caused by decreased skeletal muscle strength and mass in subjects with NAFLD. Second, systemic inflammation may also mediate a link between reduced lung function and incident NAFLD. Cigarette smoking is the most widely recognized risk factors for decreased lung function [53]. And also, other environmental exposure such as occupational dusts, chemicals, urbanization, and particulate air pollution is associated with accelerated lung function decline [40]. Inhalation of noxious particulates from environmental pollution could cause airspace inflammation and the release of pro-inflammatory cytokines (such as interleukin [IL]-6) from alveolar macrophages, which may result in damage to the airways and a decline in lung function [54]. These inflammatory cytokines could enter the circulation and stimulate systemic inflammation [54]. Indeed, increased serum CRP (product by stimulating by IL-6), a marker of systemic inflammation, has been positively associated with lung function decline, regard less of smoking status [10]. These indicate that subjects with decreased lung function may have higher exposure to various environmental insults that lead to early perturbations of lung function and, in parallel, induce a low-grade inflammatory response. Also, hypoadiponectinemia related to systemic inflammation may have a role in the development of NAFLD [55] because adiponectin has anti-inflammatory effects via inhibition of tumor necrosis factor (TNF)-α and IL-6 [56]. This raises the possibility that lung inflammation might be a marker of risk susceptibility for the development of NAFLD.

There are several strengths and limitations of our study. A major strength of our study is that we describe the association between lung function tests at baseline and incident NAFLD over 13 years of follow-up in subjects without liver fat by ultrasound examination at baseline. Another major strength of our study is its large sample size, with subjects drawn from a healthy population without overt clinical disease. There are also several limitations of our study. First, our study used ultrasonography to detect incident fatty liver as the study endpoint. We did not perform liver biopsies to diagnose NAFLD as this is not feasible in a health screening program. Therefore, since ultrasound only semi-quantitatively assesses liver fat, and not inflammation or fibrosis, we are not able to comment on associations between non-alcoholic steatohepatitis (NASH) and lung function. That being said, many population-based epidemiologic studies have diagnosed fatty liver using ultrasonography because ultrasound is recognized as a reliable tool for this purpose and has acceptable diagnostic accuracy for diagnosing steatosis [57]. Second, there is the possibility of selection bias when recruiting participants, as the study participants consisted mostly of approximately middle-aged Korean adults in an urban community who were enrolled in health promotion screening at a single university hospital. Consequently, our participants were probably healthier individuals compared with other community-based cohorts of similarly aged subjects. As a result, our findings cannot be generalized to other populations or ethnic groups. Third, there is the possibility of sampling bias among subjects who participated in the present study due to differences in socio-economic status or healthier life style, both of which could affect lung function and NAFLD. Unfortunately, this information was not available in this study. Especially, the assessment for proportion of passive smokers, more detailed data on individual’s intensity and duration of physical activity which alters the risk of NAFLD [38,45] was limited to our study. These factors may influence on our results. Finally, the present study was not hospital-based. Lack of data on the concrete environmental exposures and predisposition factors such as inflammation-sensitive plasma proteins, which could affect the susceptibility for inflammation-mediated decline of lung function [58], are also potential limitations of the present study.

In conclusion, our results showed that decreased FEV1 (%) and FVC (%) were independently associated with incident NAFLD over 13 years of follow-up. Our study is the first to describe the association between low levels of lung function at baseline and incident NAFLD. As reduced lung function at baseline is an independent risk factor for the development of NAFLD in middle-aged healthy Korean population, clinicians are aware that patients with reduced lung function are at increased risk of NAFLD regardless of their smoking status.
